# Efficacy and safety of bupropion in cancer-related fatigue, a randomized double blind placebo controlled clinical trial

**DOI:** 10.1186/s12885-020-6618-9

**Published:** 2020-02-27

**Authors:** Ebrahim Salehifar, Saeid Azimi, Ghasem Janbabai, Ehsan Zaboli, Narjes Hendouei, Fatemeh Saghafi, Samaneh Borhani

**Affiliations:** 1 Pharmaceutical Research Center, Faculty of Pharmacy, Mazandaran University of Medical Sciences, Sari, Iran; 2 Faculty of Pharmacy, Mazandaran University of Medical Sciences, Sari, Iran; 3 Gastrointestinal Cancer Research Center, Faculty of Medicine, Mazandaran University of Medical Sciences, Sari, Iran; 4 Faculty of Pharmacy, Shahid Sadoughi University of Medical Sciences, Yazd, Iran; 5 Emam Khomeini Hospital, Mazandaran University of Medical Sciences, Sari, Iran

**Keywords:** Fatigue, Cancer-related fatigue, Bupropion, Performance status, Clinical trial

## Abstract

**Background and objectives:**

Cancer-related fatigue (CRF) is one of the most prevalent complications experienced by cancer patients during and after the process of treatment. Despite conducting a lot of studies, there is no approved therapy to help manage CRF. This study aims to investigate the efficacy of bupropion on CRF.

**Materials and methods:**

In this double-blind randomized placebo-controlled clinical trial, a total of 30 eligible cancer patients suffering from fatigue were randomly divided into two groups (15 patients in each group). Bupropion was administered 75 mg/day for the first three days and 150 mg/day (divided in two doses) till the end of the study at week 6. Fatigue as the primary outcome was measured by BFI (Brief Fatigue Inventory) and FACIT-Fatigue (Functional Assessment of Chronic Illness Therapy) scales. Secondary outcomes included HADS (Hospital Anxiety and Depression Scale) and performance status (PS) measured by Karnofsky and ECOG (Eastern Cooperative Oncology Group) scales. Assessments were done at baseline, end of the second and sixth week.

**Results:**

There was no significant difference between placebo and bupropion at baseline and the end of second week. Significant difference was seen between two groups at the end of week six (*P* = 0.006 based on BFI) in favor of bupropion. In-group assessment showed improvement in fatigue levels in both groups during study time (*P* = 0.000 based on BFI for both bupropion and placebo). Secondary outcomes (e.g., HADS and PS) were not different at baseline and the end of second week. However, at the end of week six, the difference was significant in favor of bupropion.

**Conclusion:**

A six-week trial of bupropion reduces the CRF and improves the PS of cancer patients. Trial registration: Current Controlled Trials IRCT20090613002027N12, registration date: 2018-06-01.

## Introduction

Cancer-related fatigue (CRF) is one of the most common and bothersome side effects among cancer patients. It can be associated with the cancer itself, cancer treatment, and/or other symptoms such as depression or poor sleep [1]. CRF is a distressing, persistent and subjective sense of physical, emotional, and/or cognitive tiredness or exhaustion related to cancer or cancer therapy which is not proportional to recent activities and interferes with usual functioning. Compared with the fatigue experienced by healthy individuals, CRF is more severe, more distressing and less likely to be relieved by rest [2, 3].

The prevalence of CRF is not exactly estimated but some studies claim that about 59–96% of patients undergoing chemotherapy and 65–100% of patients undergoing radiotherapy will experience CRF. CRF can affect patient’s life and quality of life and because of its severity compared with usual fatigue, some patients might not continue the treatment [4–6].

The reasons of CRF occurrence and effecting factors are not clear yet but in every patient, CRF is related to dysregulation of biochemical and physiological systems of body. There are lots of studies focusing on CRF and effecting factors such as the cancer itself, treatments that patient receives (surgery, chemotherapy, radiotherapy, hormone therapy) and also chronic physical and mental situations like anemia, pain, depression, anxiety, cachexia and sleep disorders [7].

Several mechanisms including dysregulation in the function of cytokines, dysregulation in hypothalamic-pituitary-adrenal axis function, disruption in circadian rhythm, activation of vagal afferent nerve, serotonin dysregulation, changes in muscle metabolism, adenosine triphosphate dysregulation and contractile properties have been proposed for CRF [8, 9]. Dysregulation in the release of cytokines may play an important role in the development of CRF by means of inflammation. This is somehow related to chronic inflammatory processes caused by T lymphocytes [7]. Some therapeutic approaches, such as radiotherapy or chemotherapy, may also increase the levels of these cytokines [10, 11].

The HPA axis naturally regulates cortisol release which is responsible for Inhibition of cytokine release and controlling inflammation. In chronic inflammatory conditions such as cancer, which increases the production of inflammatory biomarkers, the HPA axis stimulation and cortisol activity decreases, leading to more inflammatory effects and developing fatigue [2].

Circadian rhythm disruption may cause cytokine dysregulation and developing CRF [2, 12]. Cancer can increase the release of serotonin in brain, which in turn increases the upregulation of serotonin receptors. This can lead to decrease in physical activity capacity. Activation of the vagal afferent nerve due to the release of cytokines, prostaglandins and other compounds can reduce somatomotor activity and lead to fatigue. Decreased ATP production and subsequently increased metabolic byproducts can also lead to fatigue [2, 7, 13].

Some medical and nonmedical approaches have been evaluated in CRF. L-carnitine as a medical supplement, psychological interventions and stress management plans individually or while in groups are some examples of the interventions [14, 15]. In some studies exercise and aerobic practices have been considered. Exercise can reduce CRF and is effective in improvement of muscular and breathing condition of patients [16–18]. Efficacy of exercise was more prominent in some solid tumors (breast or prostate cancer) compared with blood malignancies [19–22]. It seems that using exercise for reducing CRF needs more studies [16, 17]. Yoga, acupuncture, massage therapy and music therapy are other nonmedical methods for management of CRF [23–27].

Along with nonmedical therapies, several medical agents were investigated in reducing the fatigue in cancer patients. Herbals such as *Panax quinquefolius* [28, 29], *Withania somnifera* [30] or Chinese traditional herbals [31, 32] haven’t been able to make significant effects on CRF.

Among the chemical compounds, most studies have focused on the use of brain stimulants, blood growth factors, antidepressants, as well as progesterone [33].

Bupropion has been tested on fatigue of the cancer patients in some limited studies [34, 35]. Bupropion is a second generation antidepressant with atypical structure and has been approved for treatment of depression and smoking cessation [36]. Bupropion has been shown to be effective on fatigue of patients suffering from multiple sclerosis [37], anxiety disorders [38] and fatigue syndrome [39] and there is the possibility to be effective in CRF. Immediate-release (IR) bupropion can cause headache, insomnia, nausea/vomiting, agitation, dry mouth, constipation, tremor and seizure [40, 41].

We conducted this study to evaluate the efficacy of bupropion in CRF.

## Methods

### Patients

This randomized double-blind placebo-controlled clinical trial was done in Tooba Clinic, affiliated to Mazandaran University of Medical Sciences from September 2017 to August 2018. From 79 patients, 30 eligible patients suffering from CRF were assigned to study groups. Inclusion criteria was fatigue score of at least 4 out of 10 based on Brief Fatigue Inventory scale (BFI) [42] lasted for at least one week with no reasons. Exclusion criteria were age younger than 18, history of using CNS stimulants or antidepressants, history of epilepsy, usage of erythropoietin in the last six weeks, fatigue before cancer diagnosis, performance status score ≤ 50 based on Karnofsky performance scale, history of suicide, use of monoamine oxidase inhibitors (MAOIs) during the last two weeks, liver insufficiency (transaminases three times the upper limit of normal), kidney impairment (creatinine clearance < 60 ml/min) and pregnancy/breast feeding.

### Study design

Patients were randomly divided into two groups based on random number table: bupropion group (*n* = 15) and control group (*n* = 15). For bupropion group, bupropion was administered 75 mg/day for the first three days and 150 mg/day (75 mg/BID) for the rest of the study. Control group received placebo instead of bupropion with the same conditions. The study was six weeks long and assessments were done at baseline and end of the week two and six.

### Data gathering

Demographic and clinical information of patients were recorded at the beginning of the study. Fatigue was the primary outcome and was measured by BFI and Functional Assessment of Chronic Illness Therapy (FACIT-fatigue) scale [43]. Anxiety/depression was assessed by Hospital Anxiety and Depression Scale (HADS) and performance status (PS) was evaluated by Eastern Cooperative Oncology Group (ECOG) PS scale and Karnofsky PS scale. HADS scale is a two-section scale including 14 questions (7 for anxiety and 7 for depression) and for each questions, 0 to 3 points are considered. The score ≥ 9 for each section means that patient has anxiety or depression [44]. ECOG is a brief questionnaire consisting of five conditions (scoring from 0 to 4) that based on the patient’s status, one of these conditions will be chosen. The higher scores mean poorer performance status [45]. Karnofsky PS scale is similar to ECOG but its range of scaling is from 0 to 100 [46]. Statistical analysis was performed using SPSS software. Quantitative and qualitative variables were compared using independent t-test and chi-square test, respectively. Repeated Measure ANOVA was used to compare quantitative means in each group. All the adverse effects were categorized and recorded based on NCI common terminology criteria for adverse events [47]. Risk of seizure incidence during chemotherapy, nausea/vomiting and dry mouth were considered [48–51].

## Results

**Fig. 1:**
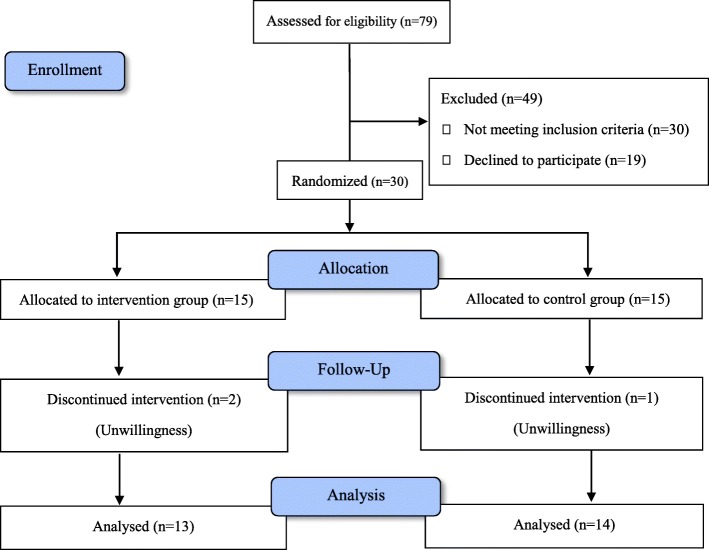
Flowchart of the study

79 patients were assessed for eligibility. 30 patients did not fulfill the study criteria and 19 declined to participate. 30 patients were randomly assigned to receive bupropion or placebo. 14 patients in control group and 13 patients in bupropion group finished the 6-week duration of the study (Fig. 1).

Demographic and clinical characteristics of patients have been shown in Table 1. The groups were not different regarding age, sex, and cancer diagnosis and treatment modalities.

**Table 1:** Demographic and clinical features of patients

		Placebo	Bupropion	*P*-value
Age (mean ± standard division)		60.21 ± 9.9	53.69 ± 12.87	0.15
Sex (number)	Male	7	4	0.31
	Female	7	9	
Diagnosis (number)	Breast cancer	5	5	0.94
	Colorectal cancer	4	3	
	Prostate cancer	2	2	
	Gastric cancer	2	1	
	Other cancers	1	2	
Other treatments (number)	Radiotherapy	7	4	0.33
	Surgery	5	4	
	Radiotherapy + surgery	2	5	

### Primary outcomes

The results of BFI and FACIT-fatigue questionnaires have been reported in Table 2. For BFI, there wasn’t any significant difference between placebo group and bupropion group at baseline (*P* = 0.3) and the end of second week (*P* = 0.8) while there was a significant difference at the end of week six (*P* = 0.006). For FACIT-fatigue scale, there were no significant differences at any periods of the study, however, the mean scores of fatigue for bupropion group were better.

**Table 2:** Mean scores of BFI and FACIT in cancer patients during the study

(A)	(B)		Mean difference (A-B)	SE	95% Confidence interval of difference		*P*-value
					Lower	Upper	
Baseline (BFI)	End of 2_nd_ week (BFI)	Placebo	0.16	0.06	0.03	0.3	0.017
		Bupropion	0.64	0.16	0.27	1	0.002
	End of 6_th_ week (BFI)	Placebo	0.49	0.1	0.27	0.7	0.001>
		Bupropion	1.8	0.17	1.8	2.2	0.001>
End of 2_nd_ week (BFI)	End of 6_th_ week (BFI)	Placebo	0.3	0.11	0.08	0.56	0.01
		Bupropion	1.16	0.14	0.8	1.4	0.001>
Baseline (FACIT)	End of 2_nd_ week (FACIT)	Placebo	- 0.57	0.7	- 2.1	0.9	0.4
		Bupropion	- 3.15	0.67	- 6.4	- 1.67	0.001
	End of 6_th_ week (FACIT)	Placebo	- 1.8	1	- 4	0.3	0.09
		Bupropion	- 7	0.9	- 9	- 5	0.001>
End of 2_nd_ week (FACIT)	End of 6_th_ week (FACIT)	Placebo	- 1.28	1.2	- 4	1.4	0.2
		Bupropion	- 3.9	0.5	- 5	- 2.7	0.001>

In Table 3, Mean difference of fatigue scores in both placebo and bupropion groups has been reported. The BFI score mean difference in all periods of time was significant for both placebo and bupropion groups. For FACIT, there was no difference between mean scores for placebo, however differences between baseline and second week and sixth week were significant for bupropion group (*P* = 0.001 and *P* > 0.001, respectively).

**Table 3:** Mean difference of fatigue scores

BFI						FACIT		
		Number	mean ± standard division	SE	*P*-value	mean ± standard division	SE	*P*-value
Baseline	Placebo	14	5.4 ± 0.6	0.16	0.3	24.6 ± 7.5	2	0.6
	Bupropion	13	5.8 ± 1.2	0.3		23.15 ± 7.2	2	
End of 2_nd_ week	Placebo	14	5.3 ± 0.5	0.14	0.8	25.2 ± 7	1.8	0.7
	Bupropion	13	5.2 ± 1.3	0.36		26.3 ± 7.3	2	
End of 6_th_ week	Placebo	14	4.9 ± 0.7	0.19	**0.006**	26.5 ± 6.8	1.8	0.15
	Bupropion	13	4 ± 0.9	0.25		30.2 ± 6.1	1.7	

### Secondary outcomes

In Table 4, mean scores of depression and anxiety have been shown. Except mean score of depression at the week 6 (*P* = 0.03), there was no difference between placebo and bupropion in case of anxiety and depression.

**Table 4:** Mean scores of hospital anxiety and depression score (HADS) during the study period

HADS (Anxiety)						HADS (Depression)		
		Number	mean ± standard division	SE	*P*-value	mean ± standard division	SE	*P*-value
Baseline	Placebo	14	8.5 ± 2.8	0.76	0.7	7 ± 3.1	0.83	0.77
	Bupropion	13	9 ± 3	0.8		5.7 ± 2.5	0.7	
End of 2_nd_ week	Placebo	14	8.2 ± 2.5	0.68	0.8	6.4 ± 2.9	0.79	0.13
	Bupropion	13	8 ± 2.7	0.77		4.7 ± 2.4	0.69	
End of 6_th_ week	Placebo	14	6.5 ± 0.6	0.7	0.22	4.7 ± 2.5	0.68	0.03
	Bupropion	13	5.3 ± 1.8	0.5		2.8 ± 1.7	0.47	

HADS_A_ Hospital Anxiety and Depression Scale (Anxiety).

HADSD Hospital Anxiety and Depression Scale (Depression).

For bupropion group, all mean differences of HADS for both anxiety and depression domains were statistically significant at the end of weeks 2 and 6 compared with baseline. Significant differences between mean scores of anxiety/depression at baseline and the end of sixth weeks (*P* = 0.009 and *P* < 0.001) and between the end of the second and sixth weeks were observed (*P* = 0.014 and *P* = 0.002) for placebo group (Table 5).

**Table 5:** Mean difference of Anxiety and Depression scores in both placebo and bupropion group

(A)	(B)		Mean difference (A-B)	SE	95% Confidence interval of difference		*P*-value
					Lower	Upper	
Baseline (HADS_A_)	End of 2nd week (HADSA)	Placebo	0.37	0.28	- 0.26	0.98	0.2
		Bupropion	1	0.3	0.3	1.7	0.009
	End of 6th week (HADSA)	Placebo	2.07	0.67	0.6	3.5	0.009
		Bupropion	3.6	0.5	2.5	4.7	0.001>
End of 2nd week (HADSA)	End of 6th week (HADSA)	Placebo	1.7	0.6	0.4	3	0.014
		Bupropion	2.6	0.4	1.7	3.48	0.001>
Baseline (HADSD)	End of 2nd week (HADSD)	Placebo	0.57	0.29	- 0.05	1.2	0.07
		Bupropion	1	0.25	0.4	1.5	0.002
	End of 6th week (HADSD)	Placebo	2.2	0.42	1.3	3.1	0.001>
		Bupropion	2.9	0.4	2	3.8	0.001>
End of 2nd week (HADSD)	End of 6th week (HADSD)	Placebo	1.64	0.4	0.7	2.5	0.002
		Bupropion	1.9	0.38	1	2.7	0.001>

HADS_A_ Hospital Anxiety and Depression Scale (Anxiety).

HADSD Hospital Anxiety and Depression Scale (Depression).

Performance status of patients was evaluated using both ECOG and Karnofsky tools (Table 6).

**Table 6:** Mean scores of performance status

ECOG						Karnofsky		
		Number	mean ± standard division	SE	P-value	mean ± standard division	SE	*P*-value
Baseline	Placebo	14	1.7 ± 0.4	0.11	0.9	67.14 ± 6.1	1.6	0.9
	Bupropion	13	1.7 ± 0.4	0.11		66.9 ± 8.5	2.3	
End of 2_nd_ week	Placebo	14	1.7 ± 0.4	0.11	0.3	67.1 ± 6.1	1.6	0.5
	Bupropion	13	1.6 ± 0.5	0.14		69.2 ± 9.5	2.6	
End of 6_th_ week	Placebo	14	1.6 ± 0.5	0.13	0.001>	70 ± 9.6	2.5	0.01
	Bupropion	13	0.9 ± 0.27	0.7		79.2 ± 7.5	2.1	

For performance status, the significant difference was seen at the end of the six weeks for placebo and bupropion groups based on both ECOG and Karnofsky scale (*P* > 0.001 for ECOG and *P* = 0.01 for Karnofsky scales, respectively). For ECOG, no difference was seen between mean scores of placebo at any time periods of study but for bupropion group, baseline and sixth weeks (*P* < 0.001) and second and sixth weeks data were significant (P < 0.001). Regarding Karnofsky scale, between baseline and the end of second week, there was no significant difference between mean scores for both groups. The most change in mean scores was for the end of week six. This change wasn’t statistically significant for placebo group while it was significantly improved in bupropion group. The change in mean scores of performance status was significant for bupropion group at the end of week six (*P* < 0.001) (Table 7).

**Table 7:** Mean difference of performance status scores

(A)	(B)		Mean difference (A-B)	SE	95% Confidence interval of difference		*P*-value
					Lower	Upper	
Baseline (ECOG)	End of 2_nd_ week (ECOG)	Placebo	0	0	0	0	0
		Bupropion	0.15	0.1	- 0.07	0.38	0.16
	End of 6_th_ week (ECOG)	Placebo	0.14	0.09	- 0.06	0.3	0.16
		Bupropion	0.8	0.1	0.6	1.07	0.001>
End of 2_nd_ week (ECOG)	End of 6_th_ week (ECOG)	Placebo	0.14	0.09	- 0.067	0.3	0.16
		Bupropion	0.7	0.13	0.4	0.98	0.001>
Baseline (Karnofsky)	End of 2_nd_ week (Karnofsky)	Placebo	0	0	0	0	–
		Bupropion	- 2.3	1.2	- 4.9	0.3	0.08
	End of 6_th_ week (Karnofsky)	Placebo	- 2.8	1.6	- 6.38	0.67	0.1
		Bupropion	- 12.3	1.2	- 14.9	- 9.6	0.001>
End of 2_nd_ week (Karnofsky)	End of 6_th_ week (Karnofsky)	Placebo	- 2.8	1.6	- 6.38	0.67	0.1
		Bupropion	- 10	1.1	- 12.4	- 7.5	0.001>

## Discussion

In this study, the efficacy of bupropion in CRF was assessed. The results showed that the more approaching to the end of study, the more effective bupropion gets and the mean scores of patients received bupropion were generally better than patients treated with placebo particularly at the end of the study. Considering BFI scores, bupropion was associated with a better efficacy compared with placebo over the time. Based on FACIT-fatigue scale, although no significant difference was seen between bupropion and placebo, the scores were higher, i.e. better, in patients on bupropion than in the placebo group. A similar result was seen in the study of Moss et al. in 2006. In their research, effects of bupropion SR on fatigue, depression and quality of life of mixed-site cancer patients was studied. 21 patients were divided into 2 groups of “depressed” and “non-depressed” and bupropion was administered for them (open-label).After one month, the assessments showed that bupropion improved the symptoms of fatigue and depression [34]. In another study performed by Cullum et al. in 2004, improvement in fatigue scores was seen two to four weeks after the beginning of the study [35]. Also in the study of Ashrafi et al. bupropion was helpful for decreasing CRF. They used 150 mg bupropion SR once a day. Based on the results of this 4 week study, bupropion showed improvement in fatigue levels of intervention group in comparison to placebo arm, however, small size of sample in the study (40 patients) was a limit to establish a strong relationship between use of bupropion and fatigue improvement. The safety of the drug during the study was reported as no seizures were seen and bupropion was well-tolerated by patients [52].

Some other agents such as methylphenidate, modafinil and donepezil have been evaluated in patients with CRF but the results of these studies were not encouraging. Siu et al. in 2013 examined the efficacy of methylphenidate in reducing CRF. Results of this study showed better improvement in CRF only in patients younger than 60 [53]. In Jean-pierre’s study, patients with mild to moderate fatigue did not show improvement whereas patients with severe fatigue did [54]. Moraska et al. reported that methylphenidate did not improve BFI and the quality of life of patients with CRF when compared with placebo, but in a subset of patients with severe fatigue and/or more advanced disease, it was helpful [55]. In an open label trial, donepezil was not superior to placebo in patients with CRF [56]. Modafinil may be a more promising drug for CRF, but in a randomized clinical trial, it was associated with only modest improvement in docetaxel-related fatigue [57]. It seems that the results of bupropion for the management of CRF in our study are hopeful.

We included HADS score and performance status as the secondary outcomes in our trial. Considering the depression domain, mean scores of depression at the end of week 6 was more favorable in patients who received bupropion. Mean scores of depression before week 6 and also anxiety scores during the whole 6 weeks of study were not significantly different between placebo and bupropion group. Other antidepressants also have been used for CRF. In a study, paroxetine 30 mg for 7 days was administered to reduce the symptoms of fatigue and depression in cancer patients. Paroxetine reduced depression but was associated with a lack of efficacy in reducing fatigue score [58]. Other agents such as coenzyme Q10 was also examined in breast-cancer patients experienced fatigue. It showed no efficacy in reducing the fatigue and depression of breast-cancer patients [59, 60].

Performance status of patients improved during the study but this improvement was more obvious in bupropion group. In both ECOG and Karnofsky scales, the most changes in scores were seen during the final week of the study. In an open-label 4-week trial, Blackhall et al. reported that modafinil improved HADS scores, CRF and ECOG performance status [61]. Improving performance status along with fatigue scores confirms the relationship between fatigue and performance of cancer patients [62] .

In our trial, the side effect profile of bupropion was acceptable. Only two patients complained of lack of appetite and insomnia and none of the patients left the study due to adverse effects. Some studies have shown that bupropion has a favorable side effect profile because almost all adverse effects reported were mild or moderate and less than 10% of patients did not continue the study due to adverse events. In addition, it does not pose the risk of abuse contrary to some agents like methylphenidate or amphetamines [34, 40, 41].

The small sample size and high dropout rate relative to the number of patients entered (10% overall and 13% in the bupropion arm) were of limitations of this study. Small sample size would influence the relationship between the use of bupropion and improvement in CRF as Ashrafi et al. mentioned in their study and also would make it difficult to identify subgroups that benefit more. The reason why some patients exited during the study was lack of desire which may show their few information about cancer therapy and because of the uncertainty over the effects of bupropion, they left the study. More studies with larger sample size are needed to be carried out to overcome these problems.

## Conclusion

Administration of bupropion 150 mg/day was effective in the management of CRF in a six-week trial. In addition to CRF, bupropion was associated with improvement in depression domain of HADS and also significantly improved performance status of cancer patients.

## Data Availability

All data generated or analyzed during this study are included in this published article.

## Abbreviations

BFI: Brief Fatigue Inventory
CRF: Cancer-Related Fatigue
ECOG: Eastern Cooperative Oncology Group
FACIT: Functional Assessment of Chronic Illness Therapy
HADS: Hospital Anxiety and Depression Scale
MAOI: Monoamine Oxidase Inhibitor
NCI: National Cancer Institute
PS: Performance status
